# Nanoencapsulated Clove Oil Applied as an Anesthetic at Slaughtering Decreases Stress, Extends the Freshness, and Lengthens Shelf Life of Cultured Fish

**DOI:** 10.3390/foods9121750

**Published:** 2020-11-26

**Authors:** Amanda Esperanza López-Cánovas, Isabel Cabas, Elena Chaves-Pozo, María Ros-Chumillas, Laura Navarro-Segura, Antonio López-Gómez, Jorge M. O. Fernandes, Jorge Galindo-Villegas, Alfonsa García-Ayala

**Affiliations:** 1 Department of Cell Biology and Histology, Regional Campus of International Excellence “Campus Mare Nostrum”, University of Murcia, 30100 Murcia, Spain; amandaesperanza.lopez@um.es (A.E.L.-C.); icabas@um.es (I.C.); 2 Centro Oceanográfico de Murcia, Instituto Español de Oceanografía, 30860 Puerto de Mazarron, Spain; elena.chaves@ieo.es; 3 Department of Agricultural Engineering, Universidad Politécnica de Cartagena, Paseo Alfonso XIII 48, 30203 Cartagena, Spain; may.ros@upct.es (M.R.-C.); laura.navarro@upct.es (L.N.-S.); 4 Regional Campus of International Excellence “Campus Mare Nostrum”, Campus Muralla del Mar, Instituto de Biotecnología Vegetal, Universidad Politécnica de Cartagena, 30202 Cartagena, Spain; 5 Faculty of Biosciences and Aquaculture, Nord University, 8049 Bodo, Norway; jorge.m.fernandes@nord.no (J.M.O.F.); jorge.galindo-villegas@nord.no (J.G.-V.)

**Keywords:** clove oil, anesthesia, stunning method, fish quality, β-cyclodextrins, inclusion complex

## Abstract

In the aquaculture industry, fish are stunned using a wide range of methods, but all of them trigger stress responses and affect the fish flesh quality. Chilled water is considered one of the most efficient methods, but even this is not a stress-free experience for the fish. Anesthetics included in the ice slurry or in water could decrease this stress and delay the loss of flesh quality. In this work, we analyze the effect of clove oil (CO) nanoencapsulated in β-cyclodextrins (β-CD) (CO + β-CD), incorporated in the stunning bath, on the stress response and the organoleptic attributes of fresh marine and freshwater fish from four economically important fish species: *Atlantic salmon*, *European seabass*, *Nile tilapia*, and *Rainbow trout*. CO + β-CD reduces the time required to induce anesthesia, independently of water salinity, habitat or water temperature. The plasmatic glucose and cortisol levels decreased in all four species, although the concentrations of CO varied between species. Moreover, plasmatic lactate level differed between the marine and freshwater fish. The use of CO + β-CD extended the shelf life of fish from all the species studied (by 3–7 days). In conclusion, using CO encapsulated in β-CD for anesthetizing fish can be regarded as an improved fish-stunning technique that reduces the anesthesia-induction time, decreases the stress response, and extends the shelf life of fresh fish.

## 1. Introduction

In aquaculture, it is well-known that fish are exposed to several levels of stress during handling and transport, and as a result of stocking density and pre-slaughter procedures [1,2,3,4]. The stress response triggers the activation of the hypothalamo–pituitary–interrenal and hypothalamus–sympathetic–chromaffin axis, which releases corticosteroids (mainly cortisol) and catecholamines into the circulatory system [4]. The release of these hormones drives metabolic and ionic changes that help fish to survive under stress conditions, but can cause alteration in the flesh quality [1].

Chilled water is frequently used for decreasing the stunning time and minimizing the physiological alterations that may occur because of the stress experienced by fish, thus improving fish welfare and the quality of the resulting product [1]. However, the immersion of fish in ice slurry is not a stress-free experience [5] and the inclusion of a proper anesthetic is necessary to decrease such stress and to minimize the induced misbalance of several biological systems.

Anesthesia is a biological state involving the partial or complete loss of sensation or loss of voluntary neuromotor control induced by chemical or non-chemical means [6]. Anesthesia rapidly induces the different stages of unconsciousness in fish, as characterized by the cessation of opercular movement and the loss of mobility [7,8,9]. In that respect, a variety of substances have been used as sedative and anesthetic in aquaculture. One of the most widely used being tricaine methanesulfonate (MS-222), which is licensed for use in food in the United States and the European Union [10]. However, MS-222 also triggers stress responses and in turn, affects fish physiology and the properties of their blood [11]. As long ago as 2005, the Canadian Council of Animal Care [12] pointed to the need to study the use of natural agents, such as clove oil (CO), as a viable alternative to chemical anesthesia.

Essential oils derived from plants have been the focus of aquaculture studies because of their diverse properties (e.g., anesthetic, antioxidant, and antimicrobial), which have been shown to reduce biochemical and endocrine alterations and consequently, improving the welfare status [4]. Among a number of natural agents, CO, in which eugenol is the main active compound, is the most common and has a reputation for being the most effective fish anesthetic [13,14]. Although CO is an effective, natural, and safe anesthetic [8], it has not been recognized by the FDA. The advantages and disadvantages of CO as a fish anesthetic have been assessed by a number of authors [8]. However, the effect of exposing fish to CO on the resulting fish flesh when consumed has not previously been studied [15]. Whatever the case, the ideal anesthetic should produce rapid anesthesia (1–5 min), and be cheap, practical to use, water soluble, and leave no residue in fish, humans, or the environment [15]. In the case of essential oils, their high volatility means that they are easily decomposed following direct exposure to heat, humidity, light, or oxygen, which would imply a decrease of their effectiveness [16]. In light of this, the use of nanotechnology has been proposed for preserving and potentiating the effects of essential oils [4].

Beta-cyclodextrins (β-CD) are naturally occurring cyclical oligosaccharides able to form solid inclusion complexes with a wide variety of hydrophobic guest molecules [17,18], while improving its solubility, increasing its stability in the presence of light, heat, and oxidizing conditions, and decreasing its volatility [17]. β-CDs are widely used in pharmaceuticals, drug delivery systems, cosmetics, and in the food and chemical industries [19]. Moreover, their low price is a strong motivation to look for new applications. Cyclodextrins may offer a safer and more efficacious option for the delivery of local anesthetics [18]. The effectiveness of CO included in β-CD (CO + β-CD) for stunning fish has been demonstrated in gilthead seabream [20]. In fact, the use of very low doses of CO + β-CD (doses of 5–15 mg/kg ice) embedded in crushed ice or ice slurry lowered several fish stress parameters in gilthead seabream compared to immersion in ice slurry alone [20].

In the present study, the effect of CO + β-CD in pre-slaughter stunning was studied in four commercially important fish species (*Atlantic salmon*, *European seabass*, *Nile tilapia*, and *Rainbow trout*), finding that the CO + β-CD reduced the time needed to induce deep anesthesia as well as the plasmatic levels of several fish stress parameters. To gain further insight into its effect on flesh quality, five sensory quality parameters were assessed in fresh fish: general aspect, eyes, gills, abdomen, and texture of muscle. It was found that a low dose of CO nanoencapsulated in β-CD improved the quality and freshness of all the species studied and extended their shelf life during ice storage.

## 2. Material and Methods

### 2.1. CO Inclusion Complex Preparation

CO (CAS number 8000-34-8) was obtained from Esencias Lozano S.A. (Esencias Lozano S.A., Caravaca de la Cruz, Spain) and β-CD (CAS number 7585-39-9) was provided by Roquette (Roquette Frères S.A., Lestrem, France). The encapsulation method used was molecular encapsulation, forming an inclusion complex, and applying the kneading method which is an efficient method to encapsulate essential oils within β-CD [21]. Briefly, 1 g of clove oil was mixed with 6.91 g of β-CD (1:1 molar ratio) in a mortar with 3 mL of ethanol, kneaded for 45 min, and finally maintained in a vacuum desiccator at room temperature for at least 72 h. This essential oil encapsulation technique in β-CD has been fully characterized with a relatively high encapsulation efficiency of 70–80% [22]. The crushed ice with CO + β-CD was manufactured by adding and dissolving the CO + β-CD powder in running water before passing it to a commercial crushed ice machine (Vogt Ice, Louisville, KY, USA) [22]. The different CO + β-CD concentrations are expressed in mg/kg (mg of CO per kg of ice crystals). Whereas the concentration of β-CD is expressed in mg/L (mg of β-CD per L of seawater or freshwater).

### 2.2. Animals and Experimental Design

Animals have been divided in two groups according to the country where were reared and slaughtered, and the guidelines used to carry out the experiments: Norway (2.2.1) or Spain (2.2.2).

#### 2.2.1. *Atlantic Salmon* (*Salmo salar*) and *Nile Tilapia* (*Oreochromis niloticus*)

The studies performed with *Atlantic salmon* and *Nile tilapia*, both reared at Nord University’s research station (Bodø, Norway), were approved by the ethics board of Nord University (Bodø, Norway). All the procedures were performed according to the conditions of the Norwegian Animal Research Authority (FOTS ID 18273).

Seventy-five specimens of *Atlantic salmon* were reared on inland tanks of 150 m^3^ of seawater at 4.0 to 6.0 °C, with a flow-through circuit, suitable aeration, and filtration system and natural photoperiod. Seventy-five specimens of *Nile tilapia* were reared in a freshwater recirculating aquaculture system at a density of 27 fish/m^3^ and fixed pH (7.6), DO (100%), temperature (28 °C), and photoperiod (13:11 LD). Besides, all animals were fed ad libitum with 0.15–0.8 mm Amber Neptun pellets (Skretting, Norway).

*Atlantic salmon* (15 fish/treatment; 2353.85 ± 85.53 g average body weight, bw) were stunned in seawater at 4.0 to 6.0 °C containing CO + β-CD at 0 (control condition) or at 40, 50, or 60 mg/L of seawater (different doses were selected in previous tests), or in seawater containing 240 mg β-CD/L (also control condition). There are two control conditions (seawater without CO + β-CD and seawater with only β-CD without CO as inclusion complex) in order to check that β-CD alone has no anesthetic effect (Table 1).

*Nile tilapia* (15 fish/treatment; 677.14 ± 28.82 g bw) were stunned in freshwater at 28.0 to 29.0 °C containing CO + β-CD (0, control condition, or 20, 40, or 60 mg/L freshwater) or β-CD (240 mg/L freshwater, control condition) (Table 1).

#### 2.2.2. European Seabass (Dicentrarchus labrax) and Rainbow trout (Oncorhynchus mykiis)

The *European seabass* and *Rainbow trout* used were handled in accordance with the guidelines of the European Union Council (2010/63/UE), Committee on the Ethics of Animal Experiments of the IEO (REGA: ES300261040017) and the approval of the Department of Water, Agriculture and Environment of the Region of Murcia Autonomous Government (Spain; A13160508).

*European seabass* specimens were bred and kept in 150 m^3^ net-tanks in the open sea of the Servicios Atuneros del Mediterráneo SL Company fish farm (San Pedro del Pinatar, Murcia, Spain). Specimens (10 fish/treatment) of 395.62 ± 57.78 g bw were stunned with crushed ice (20 mm size) mixed with seawater (ratio 1:1) at −0.2 °C, as control condition, or crushed ice containing CO + β-CD (two trials: 5 or 10 mg/kg ice or 15 or 30 mg/kg ice) (Table 1).

*Rainbow trout* specimens were kept in 300 m^3^ freshwater terrestrial tanks of Piscifactorías Andaluzas S.A. fish farm (Loja, Granada, Spain), with a flow-through circuit, suitable aeration and filtration system, and natural photoperiod. Specimens (10 fish/treatment) of 652.62 ± 31.50 g bw were stunned with crushed ice (20 mm size) mixed with freshwater (ratio 1:1) at −0.2 °C, as control group, or containing 5, 15, or 35 mg CO + β-CD/kg ice (Table 1).

In Spain, the slaughtering method of choice in the aquaculture industry is by hypothermia. To achieve the desired temperature, crushed ice at a ratio of 1:1 or 1:2 (weight/weight, *w*/*w*) is mixed with salted water collected directly from the sea or using freshwater with added salt (in the case of trout). In this study, this method was used as control with these two species.

### 2.3. Anaesthesia-Induction Stages

All specimens used in this study were fasted for 24 h before sampling. All the stunning assays developed in this study were carried out prior to killing (Table 1). The determination of the anesthesia-induction stages in each condition (see below) have been done according to the work of Keene et al. [23]. Briefly, the total loss of equilibrium stage was reached when the fish showed a total loss of muscle tone and balance, a slow, regular opercular rate, and a loss of spinal reflexes; the loss of reflex activity was defined as the total loss of reactivity, slow and irregular opercular movements are, a very slow heart rate, and the loss of all reflexes.

All the fish to be tested were placed individually in anesthetic baths, which were supplied with constant aeration. The time required to reach the desired anesthesia-induction stages were recorded using a chronometer. When the fish showed loss of reflex activity, it was removed from the anesthetic bath by a net and taken to the sampling area, where samples were taken.

For *Atlantic salmon* and *Nile tilapia*, five fish were captured from the original rearing tanks, and placed individually in the tanks (40 L or 20 L, respectively) with the different treatments used, including the control treatment. The anesthesia-induction times were assessed in the five fish simultaneously for 4 or 8 min maximum to assess the loss of equilibrium and the loss of reflex activity, respectively. This process was repeated 15 times, studying a total of 75 fish (5 fish each time × 15 times).

*European seabass* specimens were extracted directly from the floating cages located in the open sea and placed in tanks of 40 L with seawater (SW) and crushed ice (CI) at −0.2 °C containing 0 (control condition) or 5, 10, 15, or 30 mg CO + β-CD/kg ice.

*Rainbow trout* were captured from the original tanks and placed in 40-L tanks with freshwater (FW) and crushed ice at −0.2 °C containing 0 (control condition) or 5, 15, or 35 mg CO + β-CD/kg ice.

In the last two fish species, the induction times of anesthesia were observed individually (ten *European seabass* and ten *Rainbow trout* per treatment) and the observer noted the time taken by each fish to reach the above stages of anesthesia.

### 2.4. Samples Collection

The blood samples were extracted from the caudal vein, using lithium heparin-coated syringes immediately after death of the individuals. After that, plasma was obtained by centrifugation at 10,000× *g* during 5 min at 4 °C. Then, the samples were immediately frozen and stored at −80 °C until used.

### 2.5. Plasmatic Parameters

The levels of glucose (ng/mL), lactate (mmol/L), pO_2_ (mmHg), pCO_2_ (mmHg), HCO_3_ (mmol/L), TCO_2_ (mmol/L), SO_2_ (%), base excess (mmol/L), and pH were analyzed in plasma samples taken from each sampled fish, using a portable clinical analyzer (i-STAT, Abbot, Unit City, CA, USA), and the specific disposable CG4+ cartridges (Cat. # 03P85-25).

The cortisol level in fish plasma samples was analyzed by ELISA (CO103S, CALBIOTECH), according to the manufacturer’s instructions. Briefly, 25 μL of each cortisol standard solution (0, 20, 50, 100, 200, 400, and 800 ng/mL) or fish plasma sample were added to a 96-well plate. Then, 200 μL of cortisol enzyme-conjugated buffer diluted with assay diluent buffer (1:21) was added to all the wells, which were then incubated for 1 h at room temperature (20–25 °C). Finally, the wells were washed and the reaction was revealed with 100 μL of tetramethylbenzidine (TMB) enzyme substrate. The enzymatic reaction was visualized by color change and was stopped by adding 50 μL of 2 M of sulfuric acid. Absorbance was read in a spectrophotometer at 450 nm on a SPECTROstar^®^ Omega microtiter plate reader within 20 min of adding the stop solution. The absorbance values obtained were inversely proportional to the concentrations used. The concentration of cortisol was calculated according to the standard curve. All the parameters were analyzed in duplicate for each sample.

### 2.6. Sensory Evaluation

All the fish studied were packed in polystyrene boxes with crushed ice and kept refrigerated at 2 °C in cold rooms of Polytechnic University of Cartagena and Nord University, for sensory determinations and to establish their shelf life. The sensory panel consisted of seven assessors (5 female/2 males, aged 25–61) trained in fish quality evaluation following the quality index method (QIM) developed by [24] for *Nile tilapia* and by [25] for *Atlantic salmon*, *European seabass*, and *Rainbow trout*. The panelists first evaluated five quality parameters in fresh fish: general aspect, eyes, gills, abdomen, and texture of muscle, with the following QIM attributes: skin appearance (0: pearl-shiny, 1: slightly pearl shiny, yellow; mucus (0: clear, 1: milky and clotted, 2: yellow and clotted); skin odor (0: fresh seaweed, 1: neutral, 2: sour, metal, 3: rotten); eye pupils (0: clear and black, metal shiny, 1: dark grey, 2: mat, grey); eye shape (0: flat, 2: little sunken, 3: sunken); blood in abdomen (0: light red, 1: brown); odor in abdomen (0: neutral, 1: corn, 2: sour, 3: rotten); gills color (0: red, 1: light red, brown, 2: grey-brown, grey, green); gills mucus (0: transparent, 1: yellow, clotted, 2: brown); gills odor (0: fresh, seaweed, neutral, 1: metal, 2: sour, 3: rotten); texture, elasticity (0: finger mark disappears immediately, 1: finger leaves mark over 3 s). Accordingly, the provided QIM score ranges from 0 (very fresh fish) to 19 or 22 (spoiled fish) demerit points for *Nile tilapia* or *Atlantic salmon*, and *European seabass* or *Rainbow trout*, respectively. Sensory evaluation by each panelist was conducted in individual booths under controlled conditions of light (white lighting 6000 K), temperature (20–22 °C), and relative humidity (60–70%). The estimation of fish shelf life beyond the used experimentally conservation days, was performed by a linear model, (Y = aX), where Y is the QIM value, and X represents the time of ice storage [26,27], and considering the maximum acceptable QIM value equal to 65% of maximum of demerit points (spoiled fish).

### 2.7. Statistical Analysis

**Figure 1:**
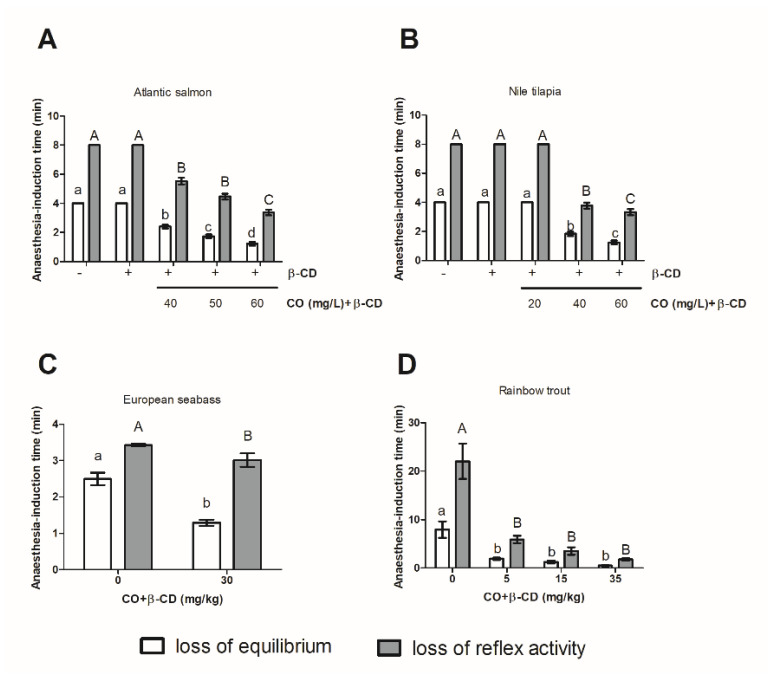
Anesthesia-induction time in *Atlantic salmon* (**A**), *Nile tilapia* (**B**), *European seabass* (**C**), and *Rainbow trout* (**D**) as a function on dose of clove oil (CO) + β-cyclodextrins (β-CD) used in stunning before slaughtering. The letters indicate statistically significant differences between groups according to one-way ANOVA and a Bonferroni post-hoc test (*p* ≤ 0.05).

**Figure 2:**
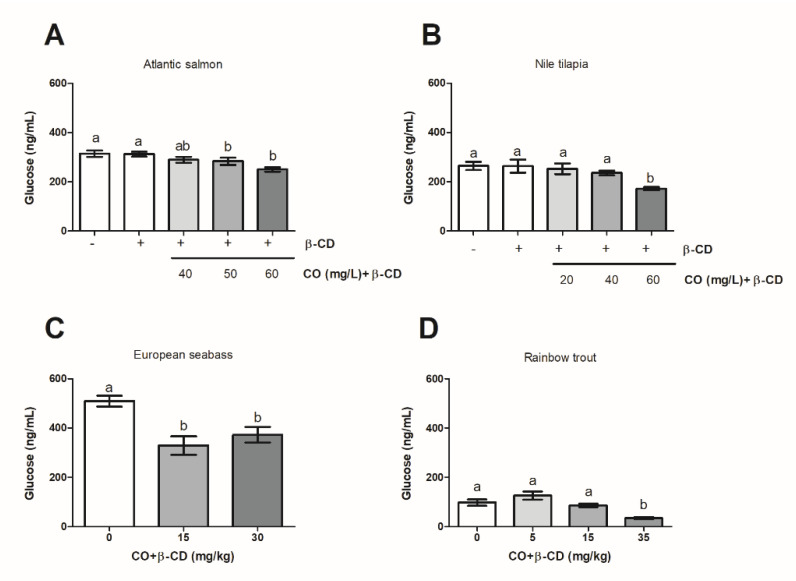
Plasmatic glucose levels in *Atlantic salmon* (**A**) and *Nile tilapia* (**B**), *European seabass* (**C**), and *Rainbow trout* (**D**) as a function on dose of CO + β-CD used in stunning before slaughtering. The letters indicate statistically significant differences between groups according to one-way ANOVA and Bonferroni post-hoc test (*p* ≤ 0.05).

**Figure 3:**
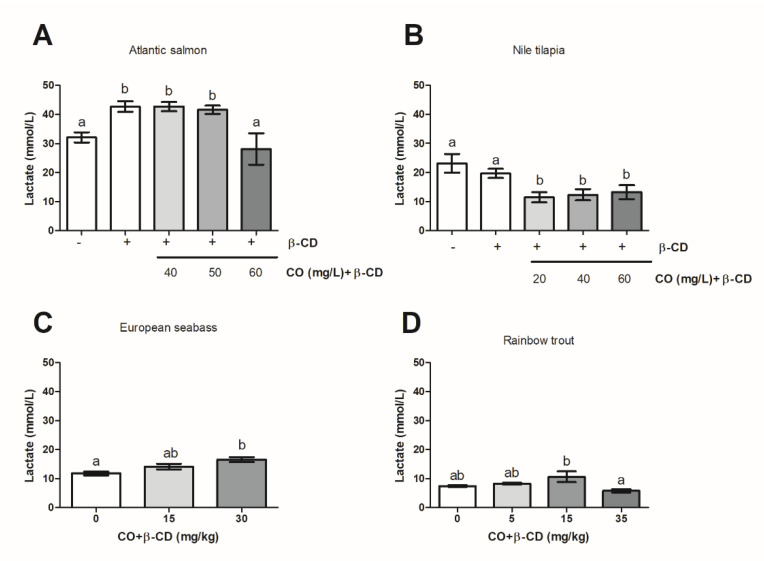
Plasmatic lactate levels in *Atlantic salmon* (**A**) and *Nile tilapia* (**B**), *European seabass* (**C**) and *Rainbow trout* (**D**) as a function on dose of CO + β-CD used in stunning before slaughtering. The letters indicate statistically significant differences between groups according to one-way ANOVA and Bonferroni post-hoc test (*p* ≤ 0.05).

**Figure 4:**
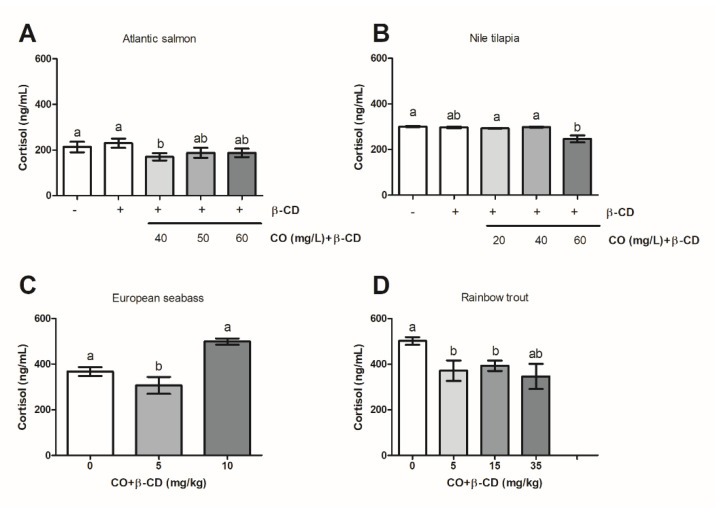
Plasmatic cortisol levels in *Atlantic salmon* (**A**) and *Nile tilapia* (**B**), *European seabass* (**C**) and *Rainbow trout* (**D**) as a function on dose of CO + β-CD used in stunning before slaughtering. The letters indicate statistically significant differences between groups according to one-way ANOVA and Bonferroni post-hoc test (*p* ≤ 0.05).

The data from the experiments, generally with three or more experimental groups, were analyzed by one-way analysis of variance (ANOVA) followed by a Bonferroni post-hoc test to determine the differences between groups. In vertical axe of Figure 1, Figure 2, Figure 3 and Figure 4 and in Tables, each value represents the mean ± S.E.M. of 10–15 fish per group in each sampling condition. The statistical significance was established at *p* ≤ 0.05. All statistical analyses were carried out using the IBM SPSS Statistic software.

## 3. Results

### 3.1. The Use of CO + β-CD Inclusion Complex Shortens the Time Needed to Induce Anesthesia

*Atlantic salmon* (Figure 1A) and *Nile tilapia* (Figure 1B) were kept for the maximum observation time (4 or 8 min) without loss of equilibrium or reflex activity, respectively, in both control groups (0 and 240 mg β-CD/L). But when CO + β-CD was incorporated in the water a decrease in both parameters was observed in both species (Figure 1A,B).

In *Atlantic salmon*, a decrease of the time to reach loss of equilibrium was correlated with the increase of CO + β-CD dose (2.39 ± 0.13 min, 1.73 ± 0.12 min, or 1.21 ± 0.12 min with 40, 50, or 60 mg/L, respectively) (Figure 1A). Concerning the loss of reflex activity, 40 or 50 mg CO + β-CD/L promoted a similar time decrease (5.53 ± 0.23 and 4.47 ± 0.19 min, respectively), while less time was needed with 60 mg/L (3.37 ± 0.19 min) (Figure 1A).

In *Nile tilapia*, a decrease of both parameters with respect to the control conditions was also observed with 40 and 60 mg of CO + β-CD/L (1.89 ± 0.14 min and 1.29 ± 0.12 min, respectively), but not when 20 mg CO + β-CD/L was used (Figure 1B).

*European seabass* specimens needed more time to achieve loss of equilibrium and loss of reflex activity in control fish (2.49 ± 0.17 and 3.42 ± 0.02 min, respectively) than in fish treated with 30 mg CO + β-CD/kg (1.28 ± 0.08 and 3.01 ± 0.10 min, respectively) (Figure 1C).

In *Rainbow trout*, a decrease in the time needed to achieve loss of equilibrium and reflex activity was also observed in fish treated with CO + β-CD (5 mg/kg, 1.92 ± 0.22 and 5.89 ± 0.80 min; 15 mg/kg, 1.27 ± 0.27 and 3.53 ± 0.17 min; and 35 mg/kg, 0.50 ± 0.10 and 1.79 ± 0.25 min, respectively) compared to the fish in the control conditions (7.95 ± 1.70 and 22.04 ± 3.66 min, respectively) (Figure 1D). However, no differences in the time needed to achieve the same anesthesia stages were observed between the different concentrations of CO + β-CD (Figure 1D).

### 3.2. The Use of CO + β-CD Inclusion Complex Decreases the Plasmatic Glucose Level

In all four species, CO + β-CD decreased the plasmatic glucose level, compared to the level of their respective control groups. A decrease was observed with 50 or 60 mg CO + β-CD/L water in *Atlantic salmon* (Figure 2A) or with 60 mg CO + β-CD/L in *Nile tilapia* (Figure 2B). No differences were observed for either species between the two control conditions assayed (Figure 2A,B). The decrease in plasmatic glucose level was attained with 15 or 30 mg CO + β-CD/kg ice in the *European seabass* (Figure 2C) or with 35 mg CO + β-CD/kg ice in *Rainbow trout* (Figure 2D), while no differences with the level of the control groups were observed at the lower concentrations (5 or 10 mg/kg ice; *European seabass*; 5 or 15 mg/kg ice, *Rainbow trout*, Figure 2D).

### 3.3. CO Included in β-CD Has Different Effects on Plasmatic Lactate Levels

The plasmatic lactate levels increased over those of their respective control groups in *Atlantic salmon* (at 40 or 50 mg CO + β-CD/L water) (Figure 3A), *European seabass* (30 mg CO + β-CD/kg ice) (Figure 3C), and *Rainbow trout* (15 mg CO + β-CD/kg ice) (Figure 3D). However, no differences from the level of their respective control groups were observed in *Atlantic salmon* at 60 mg CO + β-CD/L water (Figure 3A), *European seabass* at 5 or 10 CO mg + β-CD/L water, or at 15 mg CO + β-CD/kg ice (Figure 3C) or in *Rainbow trout* (5 and 35 mg CO + β-CD/kg ice) (Figure 3D). Interestingly, 240 mg β-CD/L water promoted a similar increase in the plasmatic levels of lactate compared with the control group (seawater at 4.0–6.0 °C) in *Atlantic salmon* (Figure 3A). By contrast, the plasmatic lactate level decreased in *Nile tilapia* treated with 20, 40, or 60 mg CO + β-CD/L water compared to the level observed in the two control groups (Figure 3B).

### 3.4. CO Included in β-CD Decreases the Plasmatic Cortisol Level

The plasmatic level of cortisol varied between the four species used: *Atlantic salmon* (214 ± 23 ng/mL) (Figure 4A), *Nile tilapia* (300 ± 2 ng/mL) (Figure 4B), *European seabass* (368 ± 20 ng/mL) (Figure 4C), and *Rainbow trout* (502 ± 17 ng/mL) (Figure 4D).

In all four fish species, the plasmatic cortisol levels decreased compared to the levels of their respective control groups when CO + β-CD was used, although the concentration needed varied.

**Figure 5:**
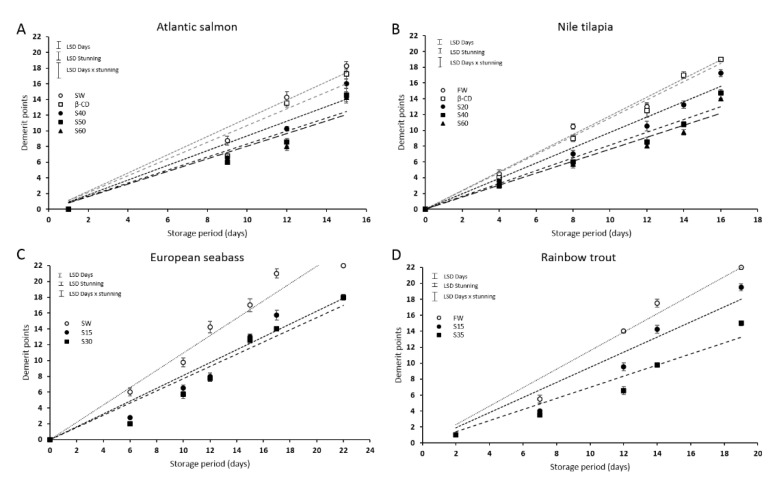
Sensory evaluation performed on fresh farmed *Atlantic salmon* (**A**), *Nile tilapia* (**B**), *European seabass* (**C**), and *Rainbow trout* (**D**) stunned using CO + β-CD or in control conditions, and then slaughtered and kept in ice at 2 °C during 15 (**A**), 16 (**B**), 22 (**C**), and 19 (**D**) days. *Atlantic salmon* treated with seawater (SW) alone or containing 240 mg β-CD/L SW (β-CD) (control conditions) or 40, 50, or 60 mg CO + β-CD/L SW (S40, S50, S60, respectively). *Nile tilapia* treated with freshwater (FW) alone or containing 240 mg β-CD/L FW (β-CD) (control conditions) or 20, 40, or 60 mg CO + β-CD/L FW (S20, S40, S60, respectively). *European seabass* and *Rainbow trout* stunned with crushed ice mixed with SW or FW, respectively (control condition) or containing 15 or 30 or 35 mg CO + β-CD/kg ice (S15, S30, S35, respectively). It is represented the observed demerit points and the corresponding linear regression fit for each treatment.

In *Atlantic salmon*, *European seabass,* and *Rainbow trout*, only the lowest concentrations of CO + β-CD/L lowered the plasmatic cortisol levels compared with their respective control groups (40 mg/L water, *Atlantic salmon*, Figure 4A; 5 mg/kg ice, *European seabass*, Figure 4C; 5 or 15 mg/kg ice, *Rainbow trout*, Figure 4D). However, at the highest concentrations (50 or 60 mg/L water, *Atlantic salmon*, Figure 4A; 10, 15 or 30, *European seabass*, Figure 4C; 35 mg/kg, *Rainbow trout*, Figure 4D), no differences were observed from the respective control group levels.

However, in *Nile tilapia* only the highest concentration used (60 mg of CO + β-CD/L) decreased to the plasmatic cortisol level to the level of the control group fish (Figure 4B) or to the levels of the groups treated with the other two concentrations assayed (20 or 40 mg of CO + β-CD/L) (Figure 4B).

### 3.5. CO + β-CD Inclusion Complex Modulates the Level of Several Other Plasmatic Parameters

As with the plasmatic level of glucose, lactate, and cortisol, there were differences between the plasmatic levels of pO_2_ (mmHg), pCO_2_ (mmHg), HCO_3_ (mmol/L), TCO_2_ (mmol/L), SO_2_ (%), base excess (mmol/L), and pH of the four fish species analyzed. Moreover modifications of these levels were observed in some stunned conditions, mainly in *Atlantic salmon* and *Nile tilapia*.

*Atlantic salmon* specimens treated with 50 or 60 mg CO + β-CD/L water (Table 2) showed an increase in the plasmatic level of pO_2_. However, the plasmatic level of HCO_3_, TCO_2_, base excess, and pH decreased in those fish compared to control fish levels (seawater alone or containing 240 mg β-CD). Interestingly, the plasmatic level of pCO_2_ was only altered when seawater containing 240 mg β-CD/L was used compared with the level of the fish treated with seawater alone. No variations were observed in the level of SO_2_ in any of the conditions assayed.

*Nile tilapia* stunned with different concentrations of CO + β-CD/L water showed differences in the levels of several plasmatic parameters (Table 2). For example, the plasmatic level of pO_2_ decreased in fish treated with 40 mg of CO + β-CD/L, while base excess level and pH increased in those fish compared, in all cases, with the control group treated with freshwater alone. Interestingly, the plasmatic level of TCO_2_ increased in fish treated with freshwater containing 240 mg β-CD/L compared with the level of the fish from the rest of the groups assayed. In addition, the base excess level in fish treated with 60 mg CO + β-CD/L was also higher than in control fish treated with freshwater alone. No variations were observed in the levels of pCO_2_, HCO_3_, and SO_2_ in any of the conditions assayed.

Few alterations were seen in the above parameters in *European seabass* (Table 3). Only the plasmatic pH was up-regulated in fish stunned with 5 and 10 mg CO + β-CD/kg, compared with the pH of the control group.

Moreover, in *Rainbow trout*, no differences were observed in any of the plasmatic parameters between fish treated with different concentrations (5, 15, and 35 mg) of CO + β-CD/kg and control fish (Table 3).

### 3.6. The Use of CO + β-CD Maintains the Sensory Attributes of Fresh Fish and Extends Shelf Life

The panelists evaluated changes in sensory scores of the four species studied during the ice storage (Figure 5). Zero points reflect the freshest state of the fish, while 19 (in salmon and tilapia) and 22 (in seabass and trout) demerit points indicate that the fish is completely spoiled. At the beginning of the storage period, panelists found typical attributes of the fresh fish in all conditions, indicating the maximum quality and freshness in all species.

Differences in the sensory characteristics were found on days 7, 9, 10, and 12 in *Rainbow trout*, *Atlantic salmon*, *European seabass*, and *Nile tilapia*, respectively. All species treated with CO + β-CD during slaughtering, regardless of the dose of CO applied, obtained better scores since they maintained the organoleptic qualities of fresh fish throughout the storage period, while the lowest scores for odor were those referring to the control samples. At the end of the storage period, the panelists rejected the control samples (slaughtered with normal ice) on days 10–12 because they showed the worst attributes: dull skin, concave eyes, opaque cornea, brown gills, and spoiled fish odor, establishing the limit of acceptability for consumption. Analyzing the shelf life prediction through linear regression of demerit points obtained during ice storage (Table 4), an extension of fish shelf life was observed for all species by five, six, six, and eight days for *Atlantic salmon*, *Nile tilapia*, *European seabass*, and *Rainbow trout*, respectively, when CO + β-CD is used as the anesthetic during slaughtering. The panelists detected no unpleasant odors or alterations in gill color and eye characteristics that were due to CO.

## 4. Discussion

In the aquaculture industry, fish are stunned using a wide range of methods depending on the species and culture conditions [28,29,30,31,32,33,34,35,36]. Among them, chilled water has been adopted as a method for decreasing the stunning time and minimizing the physiological alterations due to stress in gilthead seabream and *European seabass*, thus improving the flesh quality [1]. However, immersion of fish in ice slurry is not a stress-free experience [5], and the inclusion of an anesthetic is obligatory to reduce the stress and the induced misbalance of several biological systems. Several studies and reviews have dealt with anesthetics and fish management in aquacultural facilities [15,37], and CO has been highlighted as being the most effective natural anesthetic agent for several fish species. It is also considered acceptable for direct human consumption [15]. Although the effect of CO is dependent on fish species and size [38], the optimal dosage to induce anesthesia ranges between 50 and 100 mg/L water [8]. However, these doses are too high and can leave an unacceptable clove oil taste in the fish. β-CD improves the water solubility, chemical stability, dissolution, and release rates of various drug molecules, including natural essential oils [39,40]. In addition, encapsulation may limit the degradation/loss of some components of the essential oils, but also control their delivery at the desired time and site [21]. The increase in water solubility together with the controlled delivery time stabilizes the CO concentration throughout the anesthetic bath, speeding up the anesthesia of all the fish in the bath as they absorb the anesthetic through their gills and skin to rapidly reach the central nervous system [41]. For all that reasons, a new method of stunning based on the use of low doses of CO + β-CD inclusion complex was studied in four fish species of high economic interest in marine and continental aquaculture: *Atlantic salmon*, *Nile tilapia*, *European seabass*, and *Rainbow trout*.

Our data showed that in *Atlantic salmon* and *Nile tilapia*, the use of CO + β-CD in seawater at 4–6 °C or freshwater at 28 °C, respectively, allows the dose of CO to be reduced. Thus, *Atlantic salmon* specimens treated with 40, 50, or 60 mg CO + β-CD/L water lost their equilibrium after 2 to 3 min. However, previous studies determined the effective dose of CO for different species of salmonids as 80–100 mg/L to achieve an anesthesia time of around 3 min [42]. In the case of *Nile tilapia*, 40 mg CO + β-CD/L was the minimum dose at which the specimens achieved an anesthesia-induction time of 3.77 ± 0.20 min. Previous studies [43] proposed that the most appropriate concentration of CO to induce deep anesthesia in *Nile tilapia* is 90 mg/L, whereas we observed deep anesthesia with 40 mg CO + β-CD/L, half the dose of CO described in previous reports [43].

In fish treated under industrial farm conditions (*European seabass* and *Rainbow trout*) the use of β-CD also reduces the amount of CO needed to reach the loss of reflex activity stage in less than 3 min. Thus, previous studies fixed the optimal dose of CO as being between 40 and 100 mg/L for gilthead seabream and *European seabass* [8,9], while for *Rainbow trout* the optimal dose was around 50 mg/L [44,45]. In our study, *European seabass* and *Rainbow trout* specimens were anaesthetized with lower doses of CO (30 and 5 mg CO + β-CD/kg ice, respectively) than the doses described in previous studies for each specie [8,9,45,46]. But, why, it may be asked, are the changes produced and why do they seem to be species dependent.

In most cases, the effectiveness of anesthetics used in aquaculture depend on how it generates a stress response in fish [47]. The increase in plasmatic glucose, lactate, and cortisol has been related to such a stress response [48,49,50,51,52]. Moreover, the plasma concentration of pO_2_ tends to increase immediately after a stress response, while the plasma concentrations of pCO_2_ and HCO_3_ decrease [53]. Interestingly, the increase in glucose, lactate, and cortisol are also indicative of oxidative stress and, subsequently, tissue damage [54]. By contrast, low concentrations of HCO_3_ lead to a fall in plasma pH as the anaerobic metabolism is enhanced because of the development of hypoxia during anesthesia and it has also been related with branchial lesions [55].

In the case of *Atlantic salmon* our data showed that all the studied doses (40, 50, or 60 mg CO + β-CD/L water) decreased glucose, lactate, or cortisol plasma levels. Plasmatic glucose decreased with 50 or 60 mg CO + β-CD/L, while cortisol only decreased to control levels when 40 mg CO + β-CD/L were used. Similarly to our findings, a decrease or no effect on the plasmatic cortisol levels of *Atlantic salmon* specimens has been reported after exposure to different doses of CO [56,57]. In addition, the plasmatic level of pO_2_ increased, while HCO_3_, TCO_2_, and pH increased when 50 or 60 mg CO + β-CD/L were used, suggesting a strong stress response in these fish. However, when a 40 mg CO + β-CD/L dose was applied, none of these plasmatic parameters (pO_2_, pCO_2_, HCO_3_, TCO_2_, SO_2_, base excess and pH) was altered compared to control. All these data taken together indicate that stunning with 40 mg CO + β-CD/L induced a low stress response in *Atlantic salmon* specimens. Surprisingly, the lactate level of *Atlantic salmon* specimens increased when fish were exposed to β-CD alone and remained high even when CO was incorporated at 40 or 50 mg/L. However, salmon stunned with 60 mg CO + β-CD/L showed similar levels to the control and lower levels than fish stunned with β-CD alone, as also occurs with pCO_2_. In fact, other studies reported that the plasmatic lactate level normally increases to reach control levels upon stunning with different anesthetics including CO [58,59]. In light of the above, our data suggest that the use of β-CD with the correct dose of CO might be useful for controlling the plasmatic lactate and pCO_2_ levels during stunning in *Atlantic salmon* specimens.

In *Nile tilapia*, a reduction in the plasmatic glucose, lactate, and cortisol levels was observed when 60 mg CO + β-CD/L was used, but only the lactate level decreased to control levels at lower doses (20 or 40 mg CO + β-CD/L), as occurred with pCO_2_ and the base excess, while the pH slightly increased. Preview studies [60] also found a low glucose level in *Nile tilapia* exposed to high concentrations of menthol (75 mg/L) and eugenol (20 mg/L) compared to non-anaesthetized fish. Interestingly, a reduction in glucose in *Nile tilapia* specimens after exposure with CO (50–60 mg/L) has been described [40]. With regard to cortisol, other studies suggested that CO (30 mg/L) induces high stress levels, leading to greater mortality as a result of handling *Nile tilapia* specimens [61]. However, our data showed that 60 mg CO + β-CD/L reduced cortisol levels and pH, thus helping to lower the stress response of fish, even when there is a reduction in the plasmatic pCO_2_ level, which would be related with stress responses [53]. Interestingly, as occurs in *Atlantic salmon*, β-CD alone increased the TCO_2_ of blood in *Nile tilapia*, but the incorporation of CO reduced it to the levels seen in control fish.

Industrial farmed *European seabass* specimens treated with 15 and 30 mg CO + β-CD/kg ice showed a lower glucose level and slightly higher lactate level than the control while the cortisol level remained similar. As in *Rainbow trout*, the decrease of glucose and the increase in lactate might be due to the slower blood circulation induced by stunning [57]. But, as the cortisol level was not modified compared with the control, a stress response might occur altering the fish metabolism. This is supported by the fact that the other plasmatic parameters analyzed had similar levels to those of the control fish. However, a lower dose (5 mg CO + β-CD/kg ice) decreased the cortisol level and up-regulated the pH of blood without modifying the other plasmatic parameters, suggesting that this treatment would be optimal for *European seabass* as it reduces the stress response and preserves a normal metabolism, which, in turn, might result in improved fish welfare and a better quality of the flesh.

Also under farmed conditions, the use of 35 mg CO + β-CD/kg ice at −0.2 °C to stun *Rainbow trout* specimens led to a significant decrease in the glucose level compared to the control, while the levels of lactate and cortisol remained similar to those of the control, as did the rest of the parameters studied. In agreement with our data, other studies carried out using 30 mg/L of CO and several species of trout also found that the glucose level decreased while the rest of the stress parameters remained steady [62,63]. Interestingly, other studies performed in carp and trout with different doses of CO also found increased plasmatic glucose levels immediately after stunning [57,63,64,65]. However, when lower doses (5 and 15 mg CO + β-CD/kg ice) were used, the lactate level increased up to the control level, while the cortisol level decreased compared with the control. Taking into account that when fish are stunned with an anesthetic the circulation may decrease and the availability of oxygen in the tissues may be reduced, resulting in the production of lactate [57], our data suggest that the lowest doses of CO + β-CD applied in *Rainbow trout* might reduce the stress response of the fish since it decreased the cortisol levels, even though the lactate level increased. In fact, pO_2_, pCO_2_, HCO_3_, and the pH were not modified upon stunning with 5 mg CO + β-CD/kg ice, so the metabolic problems related to the imbalance of these parameters might be low enough to avoid irreversible lesions.

Several studies have confirmed that fish slaughter conditions affect their post-mortem quality traits such as appearance, texture, sensory qualities of fresh fish and even those of cooked fish [66,67,68]. The QIM is a demerit score system for sensory evaluation of fresh fish that can represent a linear relationship between fish freshness status and its shelf life by assessing the most descriptive quality attributes in different storage period under refrigeration conditions [24,25,27,69,70]. Linear fit to predict shelf life of fresh fish and seafoods is a method highly accepted by the scientific community [26,71,72,73] and it is considered the most appropriate method in this specific case, especially when we want to estimate the shelf life of fish beyond the used experimentally conservation days. Furthermore, the use of QIM is recommended by several European research institutes [74,75,76,77]. Based on the sensory analysis results obtained in the present study following traditional slaughter and preservation treatments, a maximum shelf life of 12.3, 10.4, 13, and 12.4 days can be achieved for *Atlantic salmon*, *Nile tilapia*, *European seabass*, and *Rainbow trout*, respectively. [69] found a shelf life of 13 days for aquacultured seabass treated with slurry ice during transportation and flake ice for storage at 4 °C. Pacheco-Rodrigues et al. (2016) reported a shelf life of 15 days for *Nile tilapia*, [78] 16 days in Lake Malawi tilapia, and [79] 13 days in whole gutted salmon stored in ice. In our experiments, all the fish species treated with CO + β-CD received better scores from the panelists compared with those given to the fish of the control treatment. The shelf life was extended to 15–17 days for *Atlantic salmon*, 12–16 days for *Nile tilapia*, 17–18 days for *European seabass,* and 15–20 days for *Rainbow trout*. Daniel et al. (2014) reported an improvement in the quality and extension of the useful life of silver catfish of up to 7 days when fish were treated with 40 μL/L of the essential oil of *Aloysia triphylla* during transport prior to slaughter. Navarro-Segura et al. [22] also reported a shelf life that increased by 4 days in the case of farmed sea bream stunned with crushed ice and using clove essential oil encapsulated in β-CD, finding a fresh odor and typical characteristics of raw fish, unlike in non-treated samples.

## 5. Conclusions

Our results showed that the use of CO + β-CD inclusion complex allows a reduction in the dose of CO necessary to induce a loss of reflex activity in several fish species treated under experimental or industrial farm conditions at different temperatures and independently of the type of water used, since it was effective in both seawater and freshwater. In addition, these lower doses of CO + β-CD allow the metabolic alteration induced by stress responses to be controlled or reduced sufficiently to confirm that the flesh quality of fish stunned/slaughtered with CO + β-CD will not be altered, the optimal dose depending on the species. In fact, our data demonstrated that the application of CO + β-CD on crushed ice (or in SW and FW) during stunning/slaughtering improved the quality and freshness of all species studied extending their shelf-life during ice storage.

## Figures and Tables

**Table 1:** Summary of the pre-slaughter stunning procedures applied to farmed *Atlantic salmon*, *Nile tilapia*, *European seabass*, and *Rainbow trout*.

	Stunning Experiments	Water Temperature (°C)	Ice Type	Sea Water:Ice Ratio (*w*/*w*)	Treatment Temperature (°C)	CO + β-CD (mg/L Water)	β-CD (mg/L Water)	CO + β-CD (mg/kg Ice)
*European seabass*	1	27.3–28.0	Crushed	1:1	−0.2	-	-	0, 5, 10
	2	27.3–28.0	Crushed	1:1	−0.2	-	-	0, 15, 30
*Rainbow trout*	1	16.0–18.0	Crushed	1:1	−0.2	-	-	0, 5, 15, 35
*Atlantic salmon*	1	4.0–6.0	n/a	n/a	4.0–6.0	0, 40, 50, 60	240	-
*Nile tilapia*	1	27.0–29.0	n/a	n/a	27.0–29.0	0, 20, 40, 60	240	-

n/a: not applicable.

**Table 2:** Plasmatic parameters level in *Atlantic salmon* and *Nile tilapia* stunned with CO + β-CD under experimental farm conditions.

	Stunning Treatments	CO + β-CD (mg/L)	Β-CD (mg/L)	pO_2_ (mmHg)	pCO_2_ (mmHg)	HCO_3_ (mmol/L)	TCO_2_ (mmol/L)	SO_2_ (%)	Base Excess (mmol/L)	pH
*Atlantic salmon*	1	0	240	203.40 ± 4.26 ^a^	29.12 ± 0.91 ^a^	9.98 ± 0.27 ^a^	10.73 ± 0.28 ^a^	100 ^a^	−19.07 ± 0.40 ^a^	7.15 ± 0.02 ^a^
				204.21 ± 2.25 ^a^	33.33 ± 0.96 ^b^	10.04 ± 0.25 ^a^	11.02 ± 0.29 ^a^	100 ^a^	−19.73 ± 0.34 ^a^	7.13 ± 0.04 ^a^
		40		204.67 ± 1.81 ^a^	30.52 ± 0.62 ^a,b^	9.86 ± 0.16 ^a^	10.73 ± 0.18 ^a^	100 ^a^	−19.53 ± 0.27 ^a^	7.12 ± 0.01 ^a^
		50		222.11 ± 2.02 ^b^	31.39 ± 0.84 ^a,b^	8.94 ± 0.18 ^b^	9.93 ± 0.18 ^b^	100 ^a^	−21.41 ± 0.29 ^b^	7.06 ± 0.01 ^b^
		60		223.30 ± 3.14 ^b^	29.79 ± 0.74 ^a,b^	8.53 ± 0.18 ^b^	9.46 ± 0.22 ^b^	100 ^a^	−21.60 ± 0.25 ^b^	7.06 ± 0.01 ^b^
*Nile tilapia*	1	0	240	200.40 ± 5.07 ^a^	17.41 ± 1.03 ^a^	8.22 ± 0.41 ^a^	8.80 ± 0.45 ^a^	100 ^a^	−18.47 ± 0.95 ^a^	7.29 ± 0.04 ^a^
				187.79 ± 1.93 ^a,b^	15.84 ± 1.68 ^a^	8.68 ± 0.39 ^a^	15.84 ± 1.68 ^b^	100 ^a^	−17.59 ± 0.43 ^a,b^	7.33 ± 0.03 ^a,b^
		20		196.62 ± 2.99 ^a,b^	14.84 ± 0.67 ^a^	8.42 ± 0.28 ^a^	8.84 ± 0.30 ^a,b^	100 ^a^	−16.92 ± 0.58 ^a,b^	7.36 ± 0.21 ^a,b^
		40		181.67 ± 7.05 ^b^	13.91 ± 1.04 ^a^	8.68 ± 0.38 ^a^	9.20 ± 0.43 ^a,b^	100 ^a^	−15.67 ± 0.71 ^b^	7.42 ± 0.03 ^b^
		60		198.43 ± 1.80 ^a,b^	16.21 ± 0.62 ^a^	9.11 ± 0.26 ^a^	9.57 ± 0.22 ^a,b^	100 ^a^	−15.54 ± 0.61 ^b^	7.63 ± 0.24 ^a,b^

The letters indicate statistically significant differences between groups according to one-way ANOVA and Bonferroni post-hoc test (*p* ≤ 0.05).

**Table 3:** Plasmatic parameters level in *European seabass* and *Rainbow trout* stunned with CO + β-CD under industrial farm conditions.

	Stunning Treatments	CO + β-CD (mg/kg)	pO_2_ (mmHg)	pCO_2_ (mmHg)	HCO_3_ (mmol/L)	TCO_2_ (mmol/L)	SO_2_ (%)	Base Excess (mmol/L)	pH
*European seabass*	1	0	234.30 ± 4.73 ^a^	17.75 ± 1.41 ^a^	5.97 ± 0.19 ^a^	6.40 ± 0.22 ^a^	100^a^	−22.91 ± 0.75 ^a^	7.13 ± 0.04 ^a^
		5	232.75 ± 4.45 ^a^	15.73 ± 1.08 ^a^	6.71 ± 0.24 ^a^	7.25 ± 0.32 ^a^	100^a^	−20.51 ± 0.45 ^a^	7.24 ± 0.02 ^b^
		10	244.71 ± 3.61 ^a^	14.97 ± 0.59 ^a^	6.51 ± 0.18 ^a^	7.10 ± 0.18 ^a^	100^a^	−20.80 ± 0.35 ^a^	7.25 ± 0.02 ^b^
	2	0	252.89 ± 7.60 ^a^	17.69 ± 1.26 ^a^	5.69 ± 0.34 ^a^	6.44 ± 0.28 ^a^	100^a^	−23.78 ± 0.97 ^a^	7.12 ± 0.05 ^a^
		15	262.75 ± 5.63 ^a^	19.81 ± 1.53 ^a^	6.21 ± 0.31 ^a^	6.75 ± 0.28 ^a^	100^a^	−23.25 ± 0.32 ^a^	7.11 ± 0.02 ^a^
		30	261.03 ± 4.97 ^a^	19.79 ± 1.08 ^a^	5.40 ± 0.14 ^a^	6.11 ± 0.24 ^a^	100^a^	−25.11 ± 0.29 ^a^	7.04 ± 0.04 ^a^
*Rainbow Trout*	1	0	271.51 ± 4.25 ^a^	30.59 ± 1.62 ^a^	11.74 ± 0.78 ^a^	12.81 ± 0.85 ^a^	100^a^	−16.51 ± 1.23 ^a^	7.19 ± 0.03 ^a^
		5	284.13 ± 5.76 ^a^	28.23 ± 2.88 ^a^	12.38 ± 0.56 ^a^	13.25 ± 0.52 ^a^	100^a^	−14.63 ± 0.96 ^a^	7.26 ± 0.04 ^a^
		15	268.88 ± 8.66 ^a^	30.01 ± 3.01 ^a^	13.41 ± 0.99 ^a^	14.51 ± 1.52 ^a^	100^a^	−15.38 ± 1.22 ^a^	7.24 ± 0.08 ^a^
		35	286.67 ± 8.69 ^a^	31.05 ± 2.21 ^a^	14.11 ± 0.70 ^a^	15.01 ± 0.73 ^a^	100^a^	−12.88 ± 1.07 ^a^	7.26 ± 0.04 ^a^

The letters indicate statistically significant differences between groups according to one-way ANOVA and Bonferroni post-hoc test (*p* ≤ 0.05).

**Table 4:** Linear regression of demerit points and estimated shelf life for *Atlantic salmon*, *Nile tilapia*, *European seabass,* and *Rainbow trout* during ice storage (at 2 °C), considering the maximum acceptable QI.

Fish Species	Stunning Treatment	Linear Regression Model	r^2^	Estimated Shelf Life (days)
*Atlantic salmon (Salmo salar)*	SW	y = 1.1608x	0.9731	12.3
	β-CD	y = 1.0690x	0.9347	13.4
	S40	y = 0.9356x	0.9319	15.3
	S50	y = 0.8312x	0.9152	17.2
	S60	y = 0.8065x	0.9065	17.7
*Nile tilapia (Oreochromis Niloticus)*	FW	y = 1.1834x	0.9897	10.4
	β-CD	y = 1.1538x	0.9881	10.7
	S20	y = 0.9738x	0.9754	12.7
	S40	y = 0.8123x	0.9634	15.2
	S60	y = 0.7611x	0.9552	16.2
*European seabass (Dicentrarchus labrax)*	SW	y = 1.0959x	0.9658	13.0
	S15	y = 0.8108x	0.9457	17.6
	S30	y = 0.7711x	0.9387	18.5
*Rainbow trout (Oncorhynchus mykiss)*	FW	y = 1.1558x	0.9658	12.4
	S15	y = 0.9477x	0.9366	15.1
	S35	y = 0.6984x	0.9297	20.5

The maximum acceptable QI is 65% of maximum of demerit points (spoiled fish), i.e., 12.35 for *Nile tilapia* and 14.3 for *Atlantic salmon*, *European seabass,* and *Rainbow trout*. y = Demerit points value; x = days of fish storage in ice; r^2^ = coefficient of regression. *Atlantic salmon* was treated with seawater (SW) alone or containing 240 mg β-CD/L SW (β-CD) (control conditions) or 40, 50, or 60 mg CO + β-CD/L SW (S40, S50, S60, respectively). *Nile tilapia* was treated with Freshwater (FW) alone or containing 240 mg β-CD/L FW (β-CD) (control conditions) or 20, 40 or 60 mg CO + β-CD/L FW (S20, S40, S60, respectively). *European seabass* and *Rainbow trout* was stunned with crushed ice mixed with SW or FW, respectively (control condition) or containing 15 or 30 or 35 mg CO + β-CD/kg ice (S15, S30, S35, respectively).
